# Protein Structure Validation and Identification from Unassigned Residual Dipolar Coupling Data Using 2D-PDPA

**DOI:** 10.3390/molecules180910162

**Published:** 2013-08-22

**Authors:** Arjang Fahim, Rishi Mukhopadhyay, Ryan Yandle, James H. Prestegard, Homayoun Valafar

**Affiliations:** 1 Department of Computer Science & Engineering, University of South Carolina, Columbia, SC 29208, USA; E-Mails: arjangvt@gmail.com (A.F.); rishi.mukhopadhyay@gmail.com (R.M.); ryandle@gmail.com (R.Y.); 2 Complex Carbohydrate Research Center, University of Georgia, Athens, GA 30602, USA; E-Mail: jpresteg@ccrc.uga.edu

**Keywords:** PDPA, RDC, unassigned, dipolar, protein, structure, model

## Abstract

More than 90% of protein structures submitted to the PDB each year are homologous to some previously characterized protein structure. The extensive resources that are required for structural characterization of proteins can be justified for the 10% of the novel structures, but not for the remaining 90%. This report presents the 2D-PDPA method, which utilizes unassigned residual dipolar coupling in order to address the economics of structure determination of routine proteins by reducing the data acquisition and processing time. 2D-PDPA has been demonstrated to successfully identify the correct structure of an array of proteins that range from 46 to 445 residues in size from a library of 619 decoy structures by using unassigned simulated RDC data. When using experimental data, 2D-PDPA successfully identified the correct NMR structures from the same library of decoy structures. In addition, the most homologous X-ray structure was also identified as the second best structural candidate. Finally, success of 2D-PDPA in identifying and evaluating the most appropriate structure from a set of computationally predicted structures in the case of a previously uncharacterized protein Pf2048.1 has been demonstrated. This protein exhibits less than 20% sequence identity to any protein with known structure and therefore presents a compelling and practical application of our proposed work.

## 1. Introduction

Considering the evolutionary mechanisms responsible for the generation of new structures in proteins, it has been speculated that there may be a limited number of unique protein folds - allowing clustering of structures into as few as ten thousand families [1]. This relatively small number has enabled a reformulation of the protein folding problem as a classification problem [2,3]. While it is clear that the folding of at least larger proteins from physical forces (force-field based folding) remains intractable and therefore outside of our computational abilities, the alternative approach of homology-based modeling (such as threading [4,5,6,7]) can easily fall within our computational reach. However, protein modeling by threading techniques is highly dependent on the availability of a comprehensive library of protein folds. Therefore, several international efforts are underway to complete a library of folds [8,9,10]. The structures deposited to the protein databank (PDB, www.rcsb.org) [11] have been very useful to classification methods and also to fragment-based methods that rely on novel structures of shorter sequences within the PDB [7,12,13,14].

**Figure 1:**
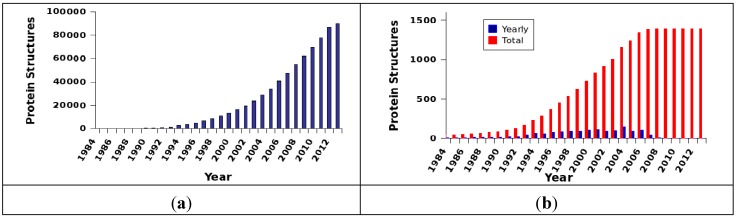
Number of protein structures in PDB (**a**) cumulative since 1974 and (**b**) unique folds reported by SCOP.

Despite the high rate with which new structures are deposited to the PDB, an analysis of these structures reveals a significant attenuation in the rate of discovery of new folds. Currently the PDB consists of over 83,000 structures of proteins representing approximately 1,400 fold families or topologies based on data published by the RCSB databank using either SCOP [15] or CATH [16] classifications. While the total number of protein structures submitted to the PDB exhibits a very productive and healthy growth [shown in Figure 1(a)], the number of newly discovered novel protein folds has been very limited over the past few years [shown in Figure 1(b)]. The high cost and the required resources can be justified for the characterization of novel protein structures; however the question remains whether more efficient ways of studying common and ordinary proteins can be established. The main contributing factor to the slow rate of discovering novel folds is the selection of target proteins solely based on sequence-homology analysis. Although this method will optimize the coverage of current protein sequence space (the space of all unique protein sequences), it may not be the optimal method of covering protein structure space. The PDB database is already populated with examples of nearly identical protein structures with dissimilar sequences. For instance, deployment of structure alignment techniques such as TALI [17] or msTALI [18] has identified groups {1LXA, 1QRE, 1TDT} or {1A17, 1E1W, 1ELR, 1E96, 1FCH, 1IHG} with sequence similarities of less than 17% and structural similarity of 2.3 Å respectively within each group. The noted inefficiency in discovery rate for novel folds by direct structure determination has motivated development of rapid and cost-effective approaches to structure determination including computational modeling of protein structures.

On other fronts, computational modeling approaches have advanced considerably in the last decade. Introduction of the Ab-Initio modeling techniques such as ROSETTA [13] and I-TASSER [5,7] during CASP VIII demonstrated the possibility of structure modeling in the absence of extended regions of sequence identity to any existing structure. Although these tools have made many significant advances, the community of structural biologists remains reluctant to completely rely on computationally modeled structures. This is partially due to the fact that modeling techniques often produce an ensemble of structures with potentially as much as 10 Å of structural diversity observed over the ensemble of the modeled structures. This high degree of inconsistency further complicates interpretation of their resultant structures. The combination of experimental data and computational modeling tools in programs such as CS-ROSETTA [19] or RDC-ROSETTA [20] have demonstrated significant improvement in eliminating some of this ambiguity. However, these tools utilize assigned NMR data, which significantly increase the data acquisition requirements of NMR spectroscopy and therefore diminish the incentive in using computational tools. It is important to note that the most time consuming and expensive portion of NMR data acquisition is related to resonance assignments. Therefore development of methods that utilize unassigned NMR data will restore the original motivation in using computational modeling tools.

Here we present two-Dimensional Probability Density Profile Analysis (*2D-PDPA)*, a significant improvement over a previously published method of validating, or identifying the structure of an unknown protein by using unassigned Residual Dipolar Coupling (RDC) data [21,22]. RDC data have been shown to be a very rich source of information about the structure and dynamics of proteins that can be acquired quickly on samples with more limited isotopic labeling. RDCs have been used in studies of carbohydrates [23,24,25], nucleic acids [26,27,28,29] and proteins [30,31,32,33,34]. The use of RDCs as the main source of structural information has led to a significant reduction in data collection and analysis, while providing the possibility of resonance assignment [35,36,37,38], and identification of dynamical regions [39,40,41]. Assigned RDC data have also been utilized in a number of instances for identification of homologous structures [32,42,43]. Another category of investigations focus on development of simultaneous assignment and structure determination from RDC data [44,45]. While these methods help in extending the frontiers of science, they do not serve as an appropriate screening tool because they either rely on enormous amounts of RDC data acquired in multiple alignment media, or assist in assignment of RDCs to an a-priori known structure. Finally from the practical standpoint, acquisition of RDC data imposes the additional requirement for successful preparation of alignment media. This issue is continually mitigated through introduction of new alignment media [46]. The large-scale applicability of RDC acquisition has been established by the Structural Genomics centers (such as NESG http://spine.nesg.org/rdc.cgi) [47], where a large fraction of their target NMR proteins (if not all) have been subjected to RDC data acquisition.

Relinquishing the need for assignment of NMR data significantly reduces the financial and temporal cost of data acquisition. Identifying a homologous structure for an unknown protein using 2D-PDPA should be of direct interest to structural biologists and pharmaceutical researchers, since they operate under the same general constraints as the structural genomic centers, which consist of reducing the cost of operation and increasing productivity. Rapid and cost effective methods of identifying protein structures, which are truly novel, could also serve to increase the general efficiency of structure determination. 2D-PDPA can also provide an optimal method of validating computationally obtained structures using a minimal set of empirical data [33,41]. This can be of benefit to pharmaceutical endeavors where researchers are often interested in validating the solution structure of a protein in the presence of a ligand relative to that of a protein, based on a structure obtained by X-ray crystallography. There are reported instances of significant structural differences between X-ray and NMR structures of the same protein (having more than 99% sequence identity). For example, structures 1HNG [48] (X-ray structure) and 1A64 [49] (NMR structure) are identical sequences but exhibit 20.9 Å of structural difference as measured over their backbone atoms. The 2D-PDPA source code has been developed in the C++ Object Oriented Programming paradigm and can be downloaded from http://ifestos.cse.sc.edu/ [50]. The current version of this program is capable of deploying on either a typical desktop environment, or Linux clusters equipped with the *qsub* scheduling protocol. 

The overall approach to validation and evaluation of the presented work consisted of three distinct tiers of experimentation with gradually increasing complexity and practical applicability. The first tier consisted of application of 2D-PDPA to a collection of proteins spanning a spectrum of sizes and structural attributes (*α*, *α*/*β*, or *β*) for which synthetic RDC data were computed. Results of this tier are used to establish a theoretical basis of the investigated mechanism under controlled conditions. The second tier consisted of application of our method to proteins with experimental RDC data. Results of this tier are used to validate the practical applicability of the proposed method. The final tier of our experiments focused on application of the proposed method to a novel protein (Pf2048.1) for which only modeled structures were available. Fitness of each computed model was determined by 2D-PDPA.

## 2. Results and Discussion

Our strategy in establishing the effectiveness of the presented work is to subject it to increasingly more challenging test cases. In the following sections we first present the performance of 2D-PDPA to test cases with simulated RDC data. The simulated cases allow for study of a method’s performance under carefully controlled conditions. It is important to note that in our studies simulated data are not error-free and they are produced to replicate the noisy experimental data as closely as possible. Following the simulated data, we present results for test cases based on experimental data that reflect the pragmatic condition. In this category, our results are first focused on instance of proteins for which both NMR and X-ray structures are known. Finally we present results of 2D-PDPA in ranking of computationally modeled structures for a target protein with no known structure.

### 2.1. Structure Identification from Simulated RDC Data

2D-PDPA was validated using synthetic data generated from eleven different protein structures (listed in Table 1) to represent a spectrum of sizes and structure types. Data from each protein structure was used to identify the correct structure from a library of 619 decoy representative structures. In each test case, the decoy structures that were not within ± 20% size of the target structure were eliminated from the pool of potential candidates. This filtering mechanism reduced the list of possible structural candidates to within 100 for proteins with less than 120 residues in length, and around 20 for larger proteins (more than 250 residues). The identification results of 2D-PDPA on the eleven randomly selected test proteins are shown in Table 1. The first column of this table lists the PDB-ID of each protein, followed by the protein size (based on number of N-H vectors), the magnitude of the uniformly added noise, and the ranking of each protein by 2D-PDPA.

**Table 1:** Results of structure identification using simulated data.

Target Structure	Size (# of NH Vectors)	Error Added	Rank
1BRF	46	±1 hz	1
1P7E	55	±1 hz	1
1SF0	67	±1 hz	1
1BQZ	75	±1 hz	1
110M	149	±1 hz	1
1NCX	160	±1 hz	1
1QHS_A	172	±1 hz	1
3FIB	241	±1 hz	1
16VP_A	289	±1 hz	1
1VSG_A	353	±1 hz	1
1A4Y_A	445	±1 hz	1

### 2.2. Structure Identification Using Experimental RDC Data

A search through the BMRB [51,52] database resulted in three proteins with backbone RDC data from two or more alignment media. These three proteins consisted of 1P7E [53], 1D3Z [54] and 1RWD [44] with backbone N-H RDC data from two alignment media. Structural homologues (both NMR and X-ray when possible) were added to our existing database of 619 decoy structures to examine 2D-PDPA’s ability to identify the actual or any homologous structures.

**Figure 2:**
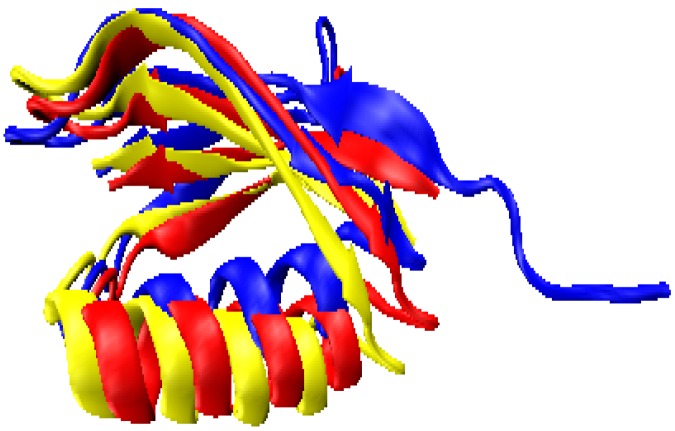
Cartoon representation of proteins 1P7E (yellow), 1IGD (blue) and 1P7F (red).

Table 2 shows the results for the protein structure 1P7E. The structure 1P7E was identified as the highest plausible structure by the 2D-PDPA as expected. Of even more interest, however, are 2^nd^ and 3^rd^ place rankings, which consisted of 1IGD and 1P7F. These are the structural homologues added to the library, and are ranked 2^nd^ and 3^rd^ respectively. The structures 1P7E, 1IGD and 1P7F exhibit around 1.0 Å of difference measured over the backbone atoms as shown in Figure 2. These results exhibit 2D-PDPA’s ability to identify not only the identical structure from a library of decoys, but also other homologous structures. Of even more importance is the fact that this experiment was performed with relatively small amounts (43 RDCs from 55 residues, 78%) of experimental data.

**Table 2:** Results of structure identification from unassigned experimental RDC data for the protein PDBID:1P7E.

Library Structure	Size (# of NH vectors)	Rank	Raw Score
1P7E	55	1	0.45
1IGD	59	2	0.47
1P7F	55	3	0.48

**Figure 3:**
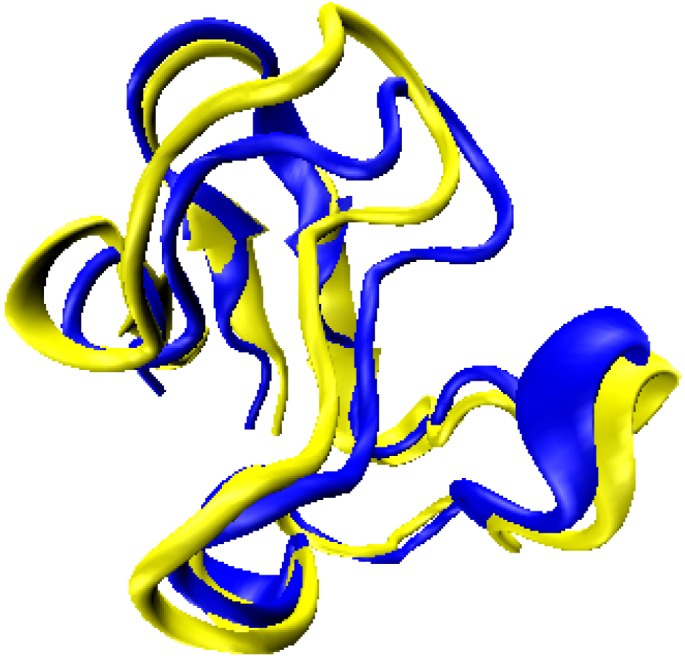
Cartoon representation of the superimposed structures 1BRF (yellow) and 1RWD (blue).

For 1RWD (results shown in Table 3), its X-Ray determined homologue 1BRF (bb-rmsd of 1.8 Å with respect to 1RWD as shown in Figure 3) ranked first. The 1RWD structure ranked second behind 1BRF. At first it may seem odd that the X-Ray structure outranked the NMR structure. However, although 2D-PDPA ranks 1BRF as the better suited structure, the ranking score of 1BRF is negligibly better than 1RWD. Furthermore, it is generally accepted that X-Ray structures fit RDC data better than NMR structures. This experiment once again demonstrates 2D-PDPA’s success in finding structural homologues within a large library of possible structures.

**Table 3:** Results of structure identification from unassigned experimental RDC data for the protein PDBID:1RWD.

Library Structure	Size (# of NH vectors)	Rank	Raw Score
1BRF	46	1	0.661
1RWD	43	2	0.667

**Figure 4:**
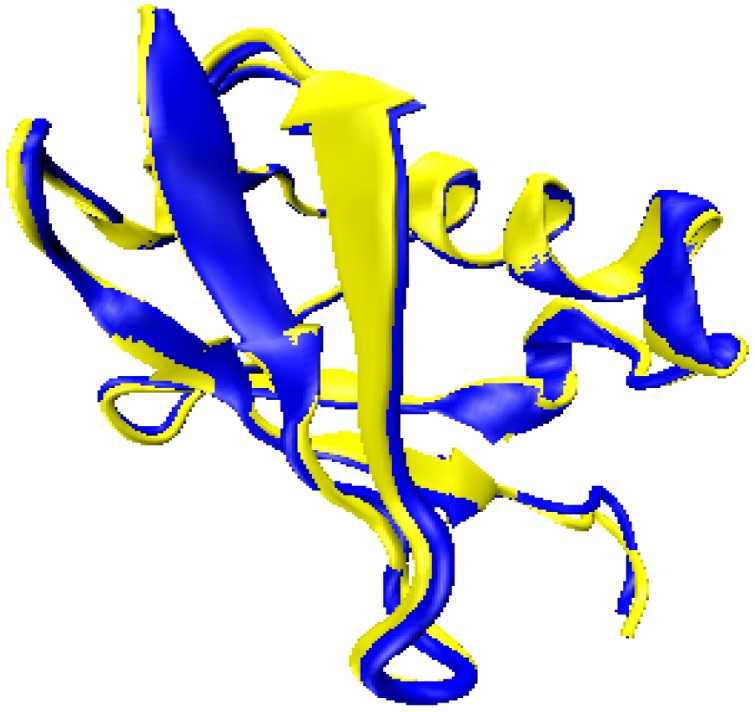
Superposition of two structures 1UBQ (blue) and 1D3Z (yellow) with bb-rmsd of 0.533 Å.

**Figure 5:**
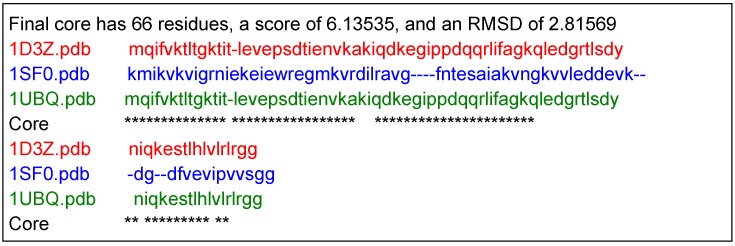
Structural alignment results of 1D3Z, 1SF0 and 1UBQ using multiple structure alignment tool msTALI.

**Figure 6:**
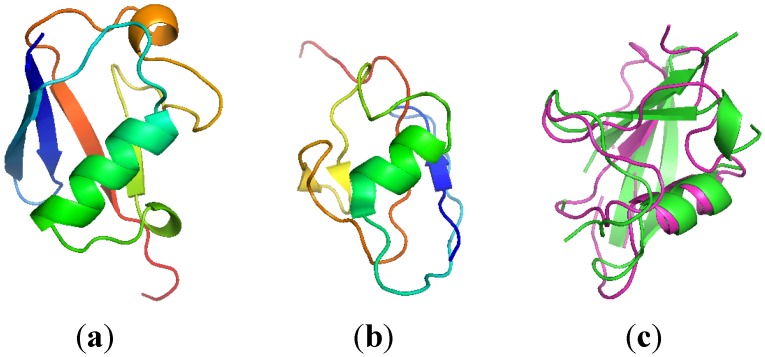
Cartoon structures of the protein 1D3Z (**panel a**) and 1SF0 (**panel b**) show no structural similarity. However msTALI identifies a 2.5 Å of bb-rmsd as shown in **panel c** (1D3Z is shown in green and 1SF0 is shown in purple).

Finally the results of structure identification for the protein 1D3Z are shown in Table 4. The NMR structure 1D3Z is ranked first, followed by its X-Ray structural homologue 1UBQ (bb-rmsd of 0.5–1.5 Å with respect to 1D3Z NMR ensemble of structures with a sample shown in Figure 4). Of additional interest is the third ranked structure 1SF0. At first glance this protein exhibits no recognizable sequence (shown in Figure 5) or structural homology with respect to 1D3Z (Figure 6(a,b)) despite the high score that is produced by 2D-PDPA. This high ranking of a seemingly unrelated protein elicited further investigations. The lack of sequence similarity is of lesser concern since there are noted instance of structural similarity in the absence of sequence similarity [17,18]. The structural similarity between 1D3Z, 1UBQ and 1SF0 was ascertained by the program msTALI [18]. Results of multiple structure alignment conducted by msTALI are shown in Figure 5. msTALI provides structural alignment that are reported in a manner similar to sequence alignment with the difference that the alignment is based on structural similarity. Results of structural alignment (shown in Figure 5) clearly indicate structural similarity over a large fraction of the three proteins with little regions of dissimilarity (indicated as gaps). The three structures exhibit 2.81 Å of similarity measured over the backbone atoms (as shown in Figure 6(c)), which indicates significant structural similarity.

**Table 4:** Results of structure identification from unassigned experimental RDC data for the protein PDBID:1D3Z.

Library Structure	Size (# of NH vectors)	Rank	Raw Score
1D3Z	72	1	0.378
1UBQ	72	2	0.450
1SF0	67	3	0.452

### 2.3. Computationally Modeled Structures of PF2048.1

**Figure 7:**
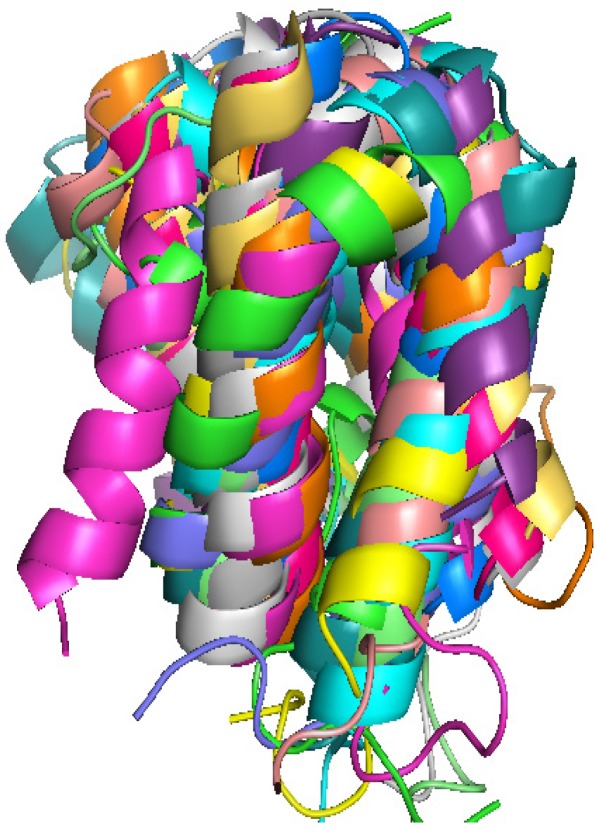
Fifteen modeled structures of PF2048.1 by ROBETTA and I-TASSER.

An ensemble consisting of ten modeled structures from ROBETTA [13,22] and five modeled structures from I-TASSER [5] for the unknown protein PF2048.1 were obtained (superimposed structures shown in Figure 7). Table 5 lists the results for an exhaustive pairwise comparison of the ensemble of fifteen structures measured over the backbone atomic positions. In this table, structures R1-R10 and I1-I5 correspond to the ROBETTA and I-TASSER structures respectively. The areas of this table that are shaded in green or yellow correspond to the intra-modeling distances, while the dark-blue areas correspond to the inter-modeling distances. Based on these results, structures modeled by ROBETTA exhibit structural similarity in the range of 2.91 Å–7.83 Å while structures modeled by I-TASSER exhibit more convergence with structural similarity in the range of 1.21 Å–3.62 Å. It is clear from this exercise that both methods have been successful in producing a reasonable model of the structure since all of them consist of a bundle of four helices. It is also clear that in the absence of a-priori knowledge of the protein’s structure, selection of the most suitable structure would have not been possible. Due to the general lack of convergence in the modeled structures, arbitrary selection of a model could lead to an erroneous structure.

**Table 5:** Pairwise backbone RMSD of the ten structures modeled by ROBETTA and five structures modeled by I-TASSER.

	R1	R2	R3	R4	R5	R6	R7	R8	R9	R10	I1	I2	I3	I4	I5
R1	0	6.51	2.93	3.01	2.95	3.39	4.33	3.37	2.73	5.42	4.43	5.14	4.82	4.25	3.48
R2	6.51	0	7.32	7.52	6.62	6.44	7.04	8.05	7.83	6.29	7.49	7.51	7.93	7.69	7.71
R3	2.93	7.32	0	4.8	5.17	3.08	6.19	4.06	3.69	4.11	6.45	7.21	7.06	6.21	5.29
R4	3.01	7.52	4.8	0	3.34	5.26	2.91	3.75	2.68	7.31	3.08	3.78	3.85	3.23	2.74
R5	2.95	6.62	5.17	3.34	0	4.72	3.1	4.04	3.94	6.81	3.32	3.78	3.66	2.96	2.83
R6	3.39	6.44	3.08	5.26	4.72	0	5.75	5.69	3.75	3.49	6.52	7	6.84	6.18	5.56
R7	4.33	7.04	6.19	2.91	3.1	5.75	0	5.45	4.2	7.73	2.89	3.02	3.56	3.22	3.43
R8	3.37	8.05	4.06	3.75	4.04	5.69	5.45	0	4.36	7.06	4.87	5.77	5.74	4.61	3.73
R9	2.73	7.83	3.69	2.68	3.94	3.75	4.2	4.36	0	6	4.92	5.57	5.25	4.77	4.04
R10	5.42	6.29	4.11	7.31	6.81	3.49	7.73	7.06	6	0	8.48	8.85	8.86	8.23	7.6
I1	4.43	7.49	6.45	3.08	3.32	6.52	2.89	4.87	4.92	8.48	0	1.21	2.75	1.31	1.91
I2	5.14	7.51	7.21	3.78	3.78	7	3.02	5.77	5.57	8.85	1.21	0	2.55	1.89	2.89
I3	4.82	7.93	7.06	3.85	3.66	6.84	3.56	5.74	5.25	8.86	2.75	2.55	0	3.07	3.62
I4	4.25	7.69	6.21	3.23	2.96	6.18	3.22	4.61	4.77	8.23	1.31	1.89	3.07	0	1.44
I5	3.48	7.71	5.29	2.74	2.83	5.56	3.43	3.73	4.04	7.6	1.91	2.89	3.62	1.44	0

### 2.4. 2D-PDPA Ranking of the Modeled Structures

**Figure 8:**
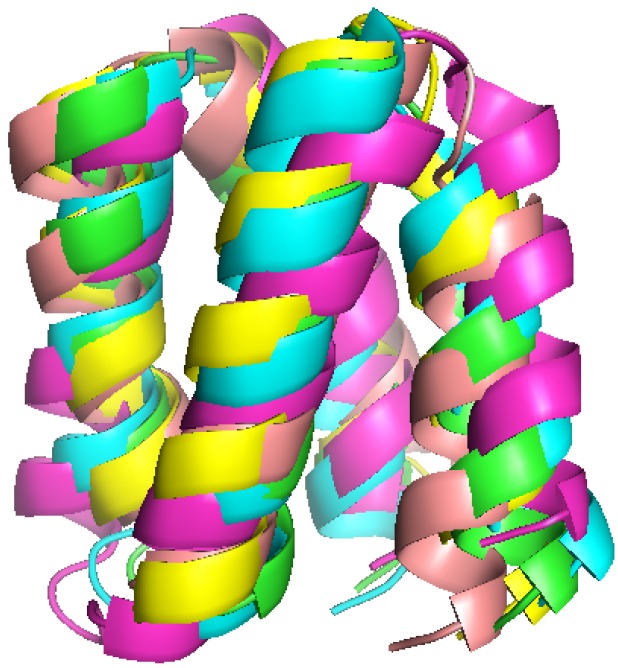
Superposition of five modeled structures (R5, R1, I5, I4, and I2) that produce 2D-PDPA scores of less than 0.80 exhibits an average 2.53 Å BB-RMSD.

2D-PDPA was applied to the ensemble of ten modeled structures of T12 by ROBETTA and five models by I-TASSER. Due to experimental conditions only 49 RDC data points were obtained from this protein in two alignment media. Considering the size of the PF2048.1 protein (79 residues), 49 RDC data points constitutes only 62% of the complete data set (38% missing data). The relative order tensors describing the alignment of this protein in each of the media were determined using the previously reported 2D-RDC [55] method (λ-map shown in Figure 15) and are listed in Table 12. Results of the 2D-PDPA ranking of ROBETTA and I-TASSER structures are shown in Table 6 and Table 7 respectively. The three columns in these tables list the structural identifiers, 2D-PDPA’s raw score for each structure, and the corrected scores respectively. The corrected scores are based on contribution of the percentage missing data on the raw score (discussed in Section 0) and are computed as shown in Equation (2). By selecting a reasonably stringent raw score of 0.8 (corrected score of 0.42) as the cutoff threshold for structural quality, the list of fifteen structures can be reduced to five; R5 and R1 of the ROBETTA structures, and I5, I4, and I2 of the I-TASSER. Figure 8 illustrates the superposition of these five structures with an average BB-RMSD of 2.53 Å. The emergence of structural convergence among the top five selected structures signifies the systematic selection mechanism of 2D-PDPA. It is important to note that 2D-PDPA’s selection mechanism is exclusively based on fitness to the experimental data and not simply based on clustering of the BB-RMSD data shown in Table 5. This independent and yet consistent selection between 2D-PDPA and BB-rmsd provides a strong evidence for accuracy of the top five structures.

**Table 6:** 2D-PDPA scores for the ten ROBETTA structures.

Modeled structure	2D-PDPA raw score	2D-PDPA corrected score
R5	0.74	0.36
R1	0.79	0.41
R8	0.81	0.43
R4	0.81	0.43
R7	0.82	0.44
R2	0.82	0.44
R6	0.83	0.45
R10	0.84	0.46
R9	0.85	0.47
R3	0.87	0.49

**Table 7:** 2D-PDPA scores for the five I-TASSER structures.

Modeled structure	2D-PDPA raw score	2D-PDPA corrected score
I5	0.73	0.35
I4	0.76	0.38
I2	0.78	0.40
I3	0.81	0.43
I1	0.82	0.44

### 2.5. Interpretation of 2D-PDPA Results for Modeled Structures of Pf2048

**Figure 9:**
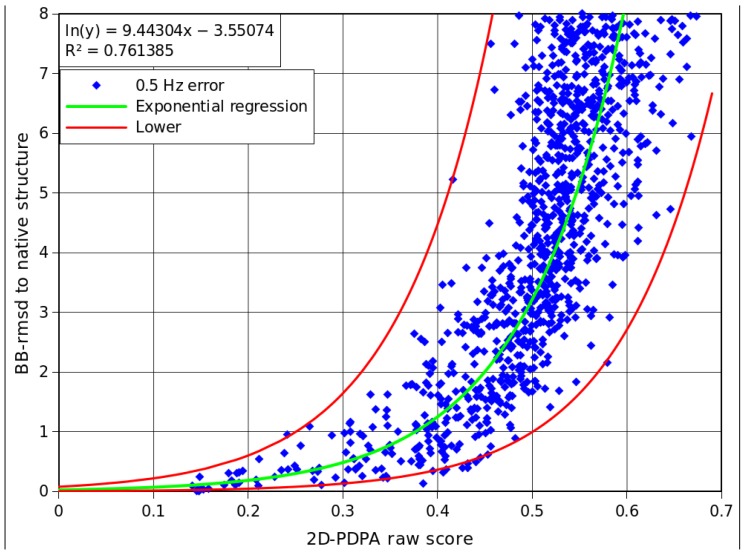
Sensitivity of 2D-PDPA analysis as a function of bb-rmsd when applied to the protein 1A1Z (83 residues).

Results listed in Table 6 and Table 7 rank the fitness of the modeled structures. However these results do not provide any information regarding the accuracy of the modeled structures with respect to the solution state structure of this protein. This information can be retrieved from further analysis of the raw scores that are provided by 2D-PDPA. To interpret the results of 2D-PDPA meaningfully, a simulation exercise has been conducted to relate the PDPA fitness score to backbone RMSD. Here we have utilized protein 1A1Z (83 residues) as a comparable structure to PF2048.1 on the basis of its size and α-helical nature. RDC data have been computed for these two proteins using typically observed order tensors as shown in Table 12. Each dataset has been corrupted through the addition of ±0.5 Hz of uniformly distributed noise. One thousand derivative structures have been generated from the native structure by randomly perturbing the backbone dihedral angles (ϕ, ψ). The set of derivative structures provided a sampling of the bb-rmsd in the range of 0–8 Å with respect to the starting structure. The 2D-PDPA procedure was then applied to the set of 1000 sample structures. Figure 9 shows the scatter plot of 2D-PDPA scores versus the backbone rmsd’s. This figure is very valuable in establishing the operational limits of 2D-PDPA as a function of data quality, and help in interpreting the results shown in Table 6 and Table 7. Based on the extrapolated upper and lower boundaries, the scores of 2D-PDPA can be converted to a range of bb-rmsd with respect to the solution state structure of the Pf2048. Table 8 lists the lower and upper estimates of bb-rmsd for each of the top five modeled structures. Therefore it can be concluded with high certainty that the R5 and the I5 structures are within 3 Å of the solution state structure of the PF2048.1.

**Table 8:** Results of 2D-PDPA analysis of modeled structures for Pf2048 with the estimated range of bb-rmsd to the solution state structure using 1A1Z as a template for the interpretation pattern.

Modeled structure	2D-PDPA corrected score	Lower bb-rmsd(Å)	Upper bb-rmsd(Å)
I5	0.35	0.22	2.72
R5	0.36	0.25	3.00
I4	0.38	0.30	3.67
I2	0.40	0.37	4.48
R1	0.41	0.41	4.95

## 3. Experimental

### 3.1. General Experimental Approach and Targeted Protein Structures

A list of protein structures that were utilized during tier 1 and tier 2 of this study are shown in Table 9. These structures range in size from 53 to 364 residues. Table 9 also shows the CATH [16] classification code for the selected structures. For structures 1NCX and 3FIB, CATH has split the sequence into two separate domains. Since 2D-PDPA uses experimental data taken from an entire structure and not an individual domain, the separate CATH domains of 1NCX and 3FIB have not been split into separate structure files. NMR experimental data was retrieved from the BMRB [51,56] for structures 1RWD [44], 1D3Z [54], and 1P7E [27].

**Table 9:** Summary of Target Structures tested with 2D-PDPA.

Structure	X-ray/NMR	# of Residues	CATH Classification	Data Source
1BRF	X-Ray	53	2.20.28.10	Synthetic
1RWD	NMR	53	2.20.28.10	Experimental
1P7E	NMR	56	3.10.20.10	Experimental & Synthetic
1D3Z	NMR	76	3.10.20.90	Experimental
1SF0	NMR	77	3.10.20.30	Synthetic
1BQZ	NMR	77	1.10.287.110	Synthetic
110M	X-Ray	154	1.10.490.10	Synthetic
1NCX	X-Ray	162	1.10.238.10	Synthetic
			1.10.238.10	
1QHS_A	X-Ray	178	3.40.50.300	Synthetic
3FIB	X-Ray	249	3.90.215.10	Synthetic
			4.10.530.10	
16VP_A	X-Ray	366	1.10.1290.10	Synthetic
1VSG_A	X-Ray	364	3.90.150.10	Synthetic
			1.10.470.10	
1A4Y_A	X-Ray	460	3.80.10.10	Synthetic

Note that structures 1BRF [57] and 1RWD are considered structural homologues with a structural similarity of 1.79 Å measured over the backbone atoms. 1BRF and 1RWD are practically the X-Ray and NMR structures of the same protein respectively.

### 3.2. Simulated and Experimental RDC Data of Target Proteins

Simulated RDC data is very important in validating the basic fundamentals and theory of any analysis. For the first phase of evaluation, synthetic RDCs for N-H vectors from 2 alignment media have been generated for each of the candidate structures shown in Table 9. Table 10 contains the order tensors used to generate the RDC data. Columns 2–6 of this table list the individual elements of the order tensors, and columns 7–8 list their corresponding axial and rhombic components of anisotropy. These order tensors have been selected to reflect alignment properties similar to other experimentally observed alignment tensors. A uniformly distributed error of ±1Hz was added to each individual data point in order to better simulate experimental conditions.

**Table 10:** Two order tensors used for generation of simulated data. These order tensors closely mimic other experimentally observed order tensors.

	Sxx	Sxy	Sxz	Syy	Syz	D_a_ (NH Hz)	R
**M1**	3.000E−4	0.0	0.0	5.000E-4	0.0	−9.45	0.25
**M2**	1.066E−04	2.367E−04	3.603E−04	−1.464E−04	4.323E−04	8.27	0.28

The atomic coordinates of the test proteins shown in Table 9 were downloaded from the PDB and the N-H vectors were extracted and stored in REDCAT format [58]. X-Ray structures were protonated using the program Reduce [59]. NMR structures that are normally reported as an ensemble of converged structures were reduced to one representative by selecting the first model in the ensemble.

The BMRB database [51,52] was screened for proteins with experimentally acquired RDC data from two or more alignment media. The list of potential protein structures was further filtered based on availability of a homologous X-ray structure in order to establish the true applicability of our approach. The final list consisted of three proteins: 1RWD [44], 1D3Z [54] and 1P7E [53]. 1P7E is the third IgG-Binding domain of protein G (GB3) (57 residues) which was refined from an X-Ray structure (1IGD) using residual dipolar couplings. 1RWD is a mutant of rubredoxin from *P. furiosis* (53 residues), which was determined entirely from residual dipolar couplings; it is structurally similar to the protein 1BRF [57] that has been characterized by X-ray crystallography. The two structures 1BRF and 1RWD exhibit a structural similarity of 1.79 Å measured over the backbone atoms.1D3Z is a 76 residue Ubiquitin protein from *Homo sapiens* and its structure was determined with carbonyl chemical shifts that were acquired by NMR spectroscopy. Structure of this protein has also been determined by X-ray crystallography, 1UBQ [57], which exhibits 0.5–1.5 Å of bb-rmsd with respect to the ensemble of NMR structure 1D3Z.

**Figure 10:**
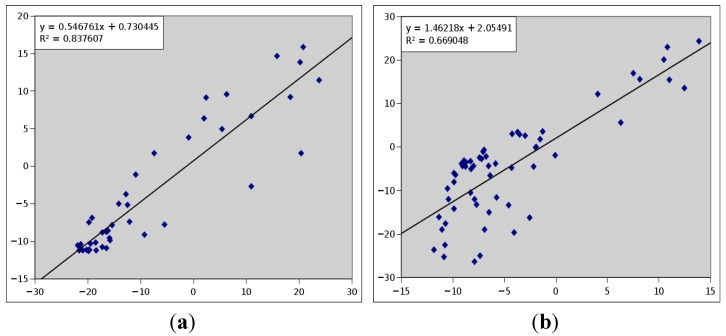
Correlation plot for the RDC data from 1P7E (**a**) and 1D3Z (**b**) in two alignment media.

Concerns can be expressed over the degree of anti-correlation that is required by 2D-PDPA between the two alignment media. Although two orthogonal and non-correlated set of data are always desirable, in practice they may not be available. To address this issue the scatter plots of RDC data in two alignment media for proteins 1P7E and 1D3Z are shown in Figure 10(a,b), respectively. The RDC data sets for proteins 1P7E and 1D3Z exhibit *R2* correlation of 0.83 and 0.67 respectively, indicating significant linear dependence between the two observed alignment media. These two datasets are presented as examples of RDC data with high degree of correlation as test cases to the 2D-PDPA method.

### 3.3. Library of Structures Representing Protein Fold Families

A library of 619 decoy structures has been used to evaluate the success of 2D-PDPA in large-scale applications. These 619 structures are the family-fold representatives of the entire PDB database in 2005 determined by FSSP (http://www.ebi.ac.uk/) [60]. Use of this library of structures was necessary for the comparison of our results to previously published work. It is important to keep in mind that although the content of the PDB database has increased significantly since 2005, the total number of distinct families of protein folds has not. SCOP [15], CATH [16], and FSSP [59] report the total number of family folds as 1393, 1233 and 2860 respectively.

The 619 structures encompass proteins ranging in size from 45 residues to over 450 residues long. Many protein structures, especially those determined by NMR spectroscopy, are reported to the PDB as an ensemble of candidate structures. In such instances, the first structure in the PDB file is used as the representative. Protein structures that had been determined by X-ray crystallography were protonated by the software package Reduce [59]. As the final step in preprocessing, atomic coordinates of the backbone N and H atoms were extracted and stored in the REDCAT [58] format. This final preprocessing step was performed to streamline our search algorithm. The 2D-PDPA software package is delivered with tools to expand the library of 619 structures, or create customized library of structures. In addition, the library of 619 libraries will soon be updated to include the complete CATH family fold representatives.

### 3.4. NMR Sample Preparation, Data Acquisition and Data

NMR sample preparation and the procedure for alignment of the Pf2048.1 with filamentous phage Pf1 has previously been described [22]. The procedure for expression and purification of the Pf2048.1 protein has also been reported previously [22]. Here we briefly highlight some of the critical aspects of this protocol and report on the data acquisition and sample preparation for a second set of RDC data in an alkyl-polyethyleneglycol (PEG) alignment medium. PF2048.1 was prepared for measurements under isotropic conditions at a concentration of 1.6 mM in 20 mM Tris and 70 mM NaCl at pH 7. All samples also contained 2 mM DTT, 0.02% azide, 1 mM DSS and 10% D_2_O. After isotropic data collection, the PF2048.1 sample was used to prepare two partially aligned samples to satisfy this requirement. A sample with Pf1 phage as the alignment medium [61] was prepared as described before [22]. A second aligned sample was prepared in 4% C_12_E_5_ (PEG, Sigma Aldrich, St. Louis, MO, USA) using previously published protocols [62]. In both cases protein samples were diluted with concentrated alignment medium in sample buffer (16% PEG, for example). Final, protein concentrations in aligned media are approximately 1.2 mM.

NMR data were collected on a Varian Unity Inova 600 MHz spectrometer at 298K using a conventional z-gradient triple resonance probe or a z-gradient triple resonance cryogenic probe (Varian Inc., Palo Alto, CA, USA). The one-bond ^1^H–^15^N couplings for isotropic and aligned samples were measured using ^15^N–IPAP–HSQC experiments [63]. Data collection included 256 t1 points, and 2048 t2 points collected over 12 h. Residual dipolar couplings were calculated as the difference of the couplings measured in the aligned and isotropic conditions. All data were processed using NMRPipe and visualized using NMRDraw [64] as previously described [22].

### 3.5. Computational Modeling of Pf2048.1

PF2048.1 is a 9.16 kDa, 78 residues; (including His-tag) monomeric protein with less than 26% sequence identity to any structurally characterized protein. A total of fifteen structural models were obtained from ROBETTA and I-TASSER modeling tools available online at http://robetta.bakerlab.org [13] and http://zhang.bioinformatics.ku.edu/I-TASSER/ [5] respectively. Both servers accept the primary sequence of a protein and return a number of modeled structures. In this instance Robetta produced 10 structural models and I-TASSER produced 5 structural models as shown in Section 0. I-TASSER and ROBETTA (a derivative of ROSETTA) are consistently highly ranked in the CASP competitions. Both of these modeling tools leverage known structural information for homologous segments of the unknown protein and perform ab-inito calculations for the remaining portions of the protein.

### 3.6. Outline of 2D-PDPA Method

**Figure 11:**
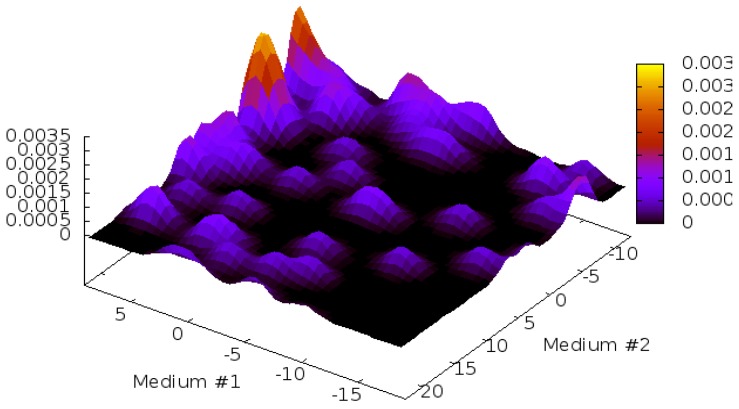
An example of a 2D-PDP map generated using kernel density estimation. This 2D-PDP can serve as a structural fingerprint.

2D-PDPA is an extension of the 1D-PDPA method [21,22] that allows simultaneous analysis of RDC data from a second alignment medium. Simultaneous consideration of RDC data from two alignment media has not previously been explored due to its computational time requirement. Our 2D-PDPA method has provided a computationally feasible approach that places a more robust system of scrutiny on the candidate structures. The overall principle that 2D-PDPA utilizes is that two similar structures must exhibit a similar distribution of RDC data as shown in Figure 11. In this figure, the distribution of RDC points is a function of the protein structure and can be used as a structural fingerprint of an unknown protein. Therefore a measure of similarity between two distributions of RDC data can be interpreted as a measure of structural similarity.

**Figure 12:**
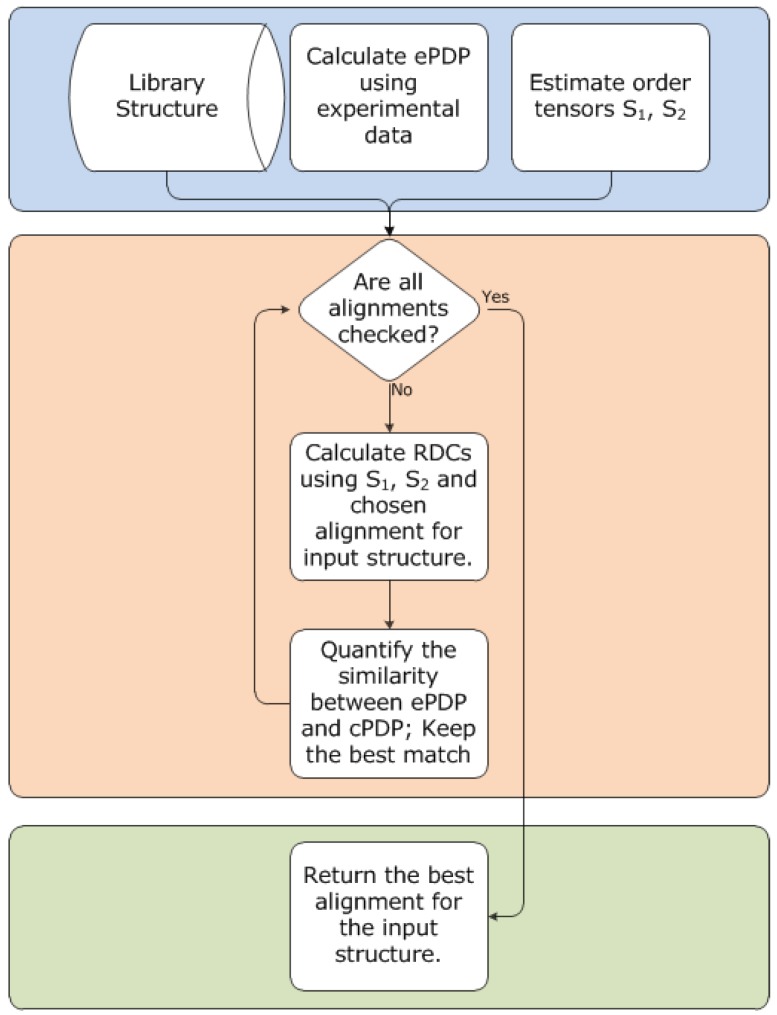
Operational schematic of the 2D-PDPA method illustrated in three main phases.

Overall operations of 2D-PDPA proceed in three main stages as shown in Figure 12. During the first stage, experimental RDC data are analyzed to estimate seven of the ten needed parameters [55,65] that are used to back-calculate RDC data from any given structure in two alignment media. During this stage, scattering of the RDC data in two alignment media is converted to a distribution function using Kernel Density Estimation [2,3,21]. This distribution is constructed through superposition of Gaussian kernels that are centered at each RDC data point. Figure 11 illustrates an example distribution map that is denoted as ePDP throughout this report. An ePDPA is referred to as a distribution map (or a fingerprint) that is generated from experimental data. During the second phase of 2D-PDPA, a similar map is created based on the back-calculated RDC data from each of the protein structures available in the library of structures using the same Kernel Density Estimation procedure. The computed maps are denoted as the cPDP’s. For each structure in the database, a cPDP is created for each possible rotation of the structure in a grid search over the Euler angles (α, β, γ) at a resolution of 5°. Each of these cPDP’s is compared to the ePDP and the best score as well as the corresponding Euler angles are recorded for each structure in the database. These 46,656 (36 × 36 × 36 rotations over α, β and γ) alternate cPDP’s are created as a result of a 5° grid search over the three remaining parameters that are needed for back-calculation of the RDC data. These three remaining parameters essentially represent all possible orientations of any given structure. RDCs are insensitive to 180° rotations; hence the search space can be reduced to a range of [0°–180°] in increments of 5° for each parameter. The best matching score and its corresponding three search parameters are recorded for the third and final stage of 2D-PDPA. During the concluding stage of the 2D-PDPA, all of the proteins in the library of structures are ranked based on their 2D-PDPA fitness, which was measured during previous stage, and the results are reported.

### 3.7. Scoring and Interpretation of 2D-PDPA Raw Scores

In contrast to 1D-PDPA [21,22] that utilizes *χ*^2^ metric [3] of comparison, 2D-PDPA employs a more intuitive Manhattan (or City-Block) metric [3] for comparison of *cPDP* and *ePDP*. Equation (1) describes the Manhattan distance that is computed by 2D-PDPA. In this equation *B* denotes the 2D-PDPA’s raw score (Block score), the summation indices *i* and *j* traverse the entire range of RDCs over the two alignment media *M_1_* and *M_2_*, and *δ_i_* and *δ_j_* denote the step size of uniform grid sampling along each of the RDC dimensions. In this equation *cPDP_ij_* and *ePDP_ij_* represent the likelihood reported by each *PDP* set at locations *i* and *j*. Since the cPDP and ePDP are normalized to be a qualified probability density functions, their integral over the entire range of RDCs equates to one. Therefore the B-score will have an effective range of [0–2], where a score of 0 indicates 100% similarity and a score of 2 indicates 0% similarity between the two structures. Furthermore, when the B-score is normalized by a factor of ½, it can be interpreted as a fraction of structural dissimilarity between the query protein and the unknown target protein. This mechanism of interpretation can be used in establishing a threshold for acceptability of a ranked sample structure. A number of factors such as: quality of the experimental data and completeness of data need to be considered in interpretation of the B-scores. However unlike utility of assigned RDC data, normalization based on strength of alignment (such as Q-factor) is not needed. This is due to the fact that 2D-PDPA is based on comparison of distribution of RDCs and not direct comparison of RDCs:

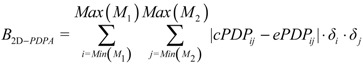
(1)

In instances where meaningful bb-rmsd values can be calculated (such as the Pf2048 exercise) between the members of the search database and the unknown protein, a more informative relationship between the 2D-PDPA’s B-score and the expected bb-rmsd can be established. Such interpretation patterns can be created based on the following observations:

1. Interpretations patterns are primarily a function of class of protein structure (α of β protein) and protein size
2. Interpretation patterns depend on completeness of data
3. Interpretation patterns exhibit a dependency on quality of experimental data, and more directly on the quality of the two estimated order tensors

**Figure 13:**
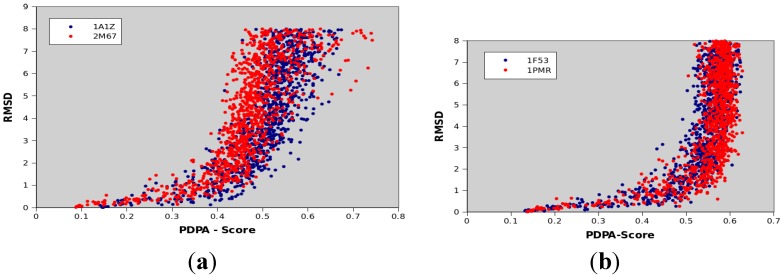
Sensitivity of 2D-PDPA analysis as a function of bb-rmsd when applied to (**a**) two unrelated a-proteins and (**b**) two unrelated b-proteins. Simulations included addition of ±0.5 Hz of uniformly distributed noise.

The latter dependency is intuitive and is investigated in the literature [21,22,55,65] and it is therefore not discussed further in this report. We demonstrate the first and second above dependencies by generating a scatter plot of bb-rmsd versus their corresponding B-score for 1000 derivative structures. These derivative structures were generated by randomly altering backbone dihedral angles of the native structure for a given protein. The ensemble of altered structures was used to compute a B-score and bb-rmsd with respect to the native structure. In this exercise we have used two sample α-proteins (1A1Z and 2M67) and two sample β-proteins (1F53 and 1PMR) that are approximately of equal sizes. Table 11 shows the detailed information for each of these four proteins. It is important to note that the two proteins in each structural class are unrelated. Figure 13 illustrates the interpretation patterns for each of the two classes. The two patterns are remarkably well conserved between the two proteins from the same structural class. Any noted differences are due to random sampling of the space and will be resolved by increasing the number of random sampling.

**Table 11:** List of four proteins that are used in establishing the properties of 2D-PDPA bb-rmsd interpretation patterns.

Protein PDBID	Protein Size	CATH Classification	Number of Secondary Structural Elements
1A1Z	83	1.10.533.10	11 α-helices
2M67	81	Not available yet	6 α-helices
1F53	84	2.60.20.30	6 β-strands
1PMR	80	2.40.50.100	6 β-strands

**Figure 14:**
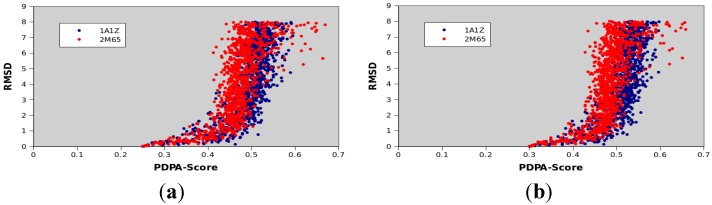
Sensitivity of 2D-PDPA analysis as a function of bb-rmsd on two α-proteins 1A1Z and 2M67 (**a**) with 25% of the data randomly removed and (**b**) with 30% of the data randomly removed.

These interpretation patterns also exhibit a very predictable behavior as a function of missing data. To illustrate this point, we performed a similar exercise as above on the α-protein set {1A1Z, 2M65} by randomly removing 25% and 30% of the data. The final results are shown in Figure 14 and as expected, the lowest scores correspond to the percentage of missing data [shown in Equation (2)]. This exercise was repeated for a number of other proteins with very similar results (not shown here). Based on this observation, a corrected score can be computed by subtracting the fraction of missing data from the raw score. This correction eliminates the contribution of missing data and allows for easier comparison of 2D-PDPA’s scoring mechanism across different instances of analyses:

*Corrected Store = Raw Score − Missing Data* (2)

The noted properties of the 2D-PDPA’s Block scoring mechanism enables creation of an interpretation pattern from another protein with similar structural attributes as the target protein. The resultant interpretation pattern can then be used to establish the quality of the 2D-PDPA’s selected structure. We have utilized this mechanism to establish the quality of modeled structures for Pf2048.

### 3.8. Computational Facilities

Large-scale applications of 2D-PDPA require execution times that exceed 24 h when implemented on a typical desktop computer. To expedite our data analysis, 2D-PDPA has been ported and utilized on a Linux cluster. This high-performance computing platform consisted of a 76-node 152-core Intel Xeon (3.4 GHz)/ Dell distributed cluster with 4GB of local memory per CPU, and a total of 1TB of storage space. The backbone inter-CPU communication is facilitated by Topspin Infiniband Interconnect. Full deployment of 2D-PDPA with a library of 619 structures at 5° resolution of search can be completed in approximately one hour.

## 4. Theoretical Background

### 4.1. Residual Dipolar Coupling

Residual Dipolar Couplings (RDCs) are obtained from the Nuclear Magnetic Resonance Spectroscopy (NMR) of weakly aligned samples. Although RDCs had been observed as early as 1963 [66] in nematic environments, they have only recently become more commonly used for direct investigation of molecular structures and internal dynamics. RDCs have been the subject of a number of reviews [46,67]. They have been used by the community of investigators in application to structure determination of proteins [33,34,68,69,70,71,72], nucleic acids [71,73,74,75,76] and carbohydrates [77,78,79,80]. RDCs have been utilized in structure determination of challenging proteins such as membrane proteins [81,82,83,84], homo-oligemeric proteins [70] and in the study of dynamics [75,85,86]. RDCs have also been used in the assembly of molecular complexes from individual domains [78]. Residual Dipolar Couplings are a measurement of a dipole-dipole interaction between two spin systems as aligned in the external magnetic field of an NMR instrument. Equation (3) shows the time-average formulation of the RDC phenomenon for two spin ½ nuclei. Here, i and j denote the two nuclei and θ is the angle between the inter-nuclear vector and the magnetic field. The angle brackets in Equation (3) denote a time *averaging,* and D_max_ is the maximum observable RDC value for a particular pair of nuclei as defined in Equation (4):

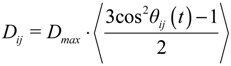
(3)

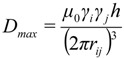
(4)

In Equation (4), *µ_0_* is the magnetic permeability of free space, *i* and *j* are the gyro-magnetic ratios of the two corresponding nuclei, *r* is the distance of the inter-nuclear vector between *i* and *j* and *ħ* is the normalized Planck’s constant. For isotropically tumbling molecules, the time-average observed RDC value from Equation (3) is reduced to zero. Observation of the RDC interaction, therefore, requires perturbation of the isotropic tumbling of molecules by introducing an alignment medium. Examples of alignment media include liquid crystalline bicelles, filamentous bacteriophage (Phage), polyacrylamide gel (PAG), and alkyl-polyethylene glycol (PEG) detergents in water [65,87] to name a few. The use of such alignment media induces an anisotropic distribution of orientations for tumbling molecules, therefore allowing for non-zero RDC values to be observed. Subsuming the effects of time averaging and proper factorization of the RDC interaction produces the final formulation of the RDC interaction as shown in Equation (5).


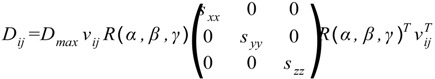
(5)


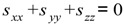
(6)

In Equation (5), *v_ij_* is the unit inter-nuclear vector between atoms *i* and *j*, and *R(α, β,γ)* is an Euler rotation (defined by the three angles α, β and γ) that describe the preferred alignment of the structure with relation to any arbitrary orientation of the protein. The preferred orientation of the molecule within the NMR magnetic field is referred to as the Principal Alignment Frame (PAF). The principal order parameters *s_xx_*, *s_yy_* and *s_zz_* describe the degree of order (or strength of alignment) along each of the main axes of alignment. Equation (5) can be used in various contexts [21,33,34,55,58,65], however within the scope of this work it has been used to back-calculate RDC data for a given structure and estimate principal order parameters *s_xx_*, *s_yy_* and *s_zz_*.

**Figure 15:**
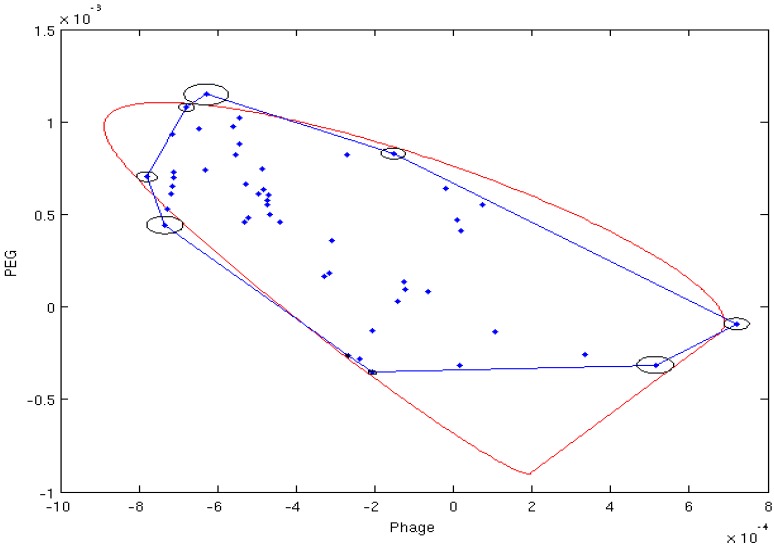
Results of 2D-RDC analysis based on unassigned data from Pf2048.1 obtained in phage and PEG alignment media. The blue lines indicate the convex hull of the 2-D RDC data set determined from the experimental data and the red line indicates the convex hull of the distribution of 2D-RDC data points for the order tensor estimate.

### 4.2. Estimation of Order Tensor and Calculation of RDCs

Back calculation of RDC data for a given structure is of central importance to the work that is presented here. Equation (5) can be used to conveniently back calculate RDC data for a given protein structure in the presence of additional information. In Equation (5), the coordinates for any interacting vector v_ij_ can be obtained from a given PDB file. Five additional parameters [α, β, γ, s_yy_, s_zz_; note that s_xx_ can be reconstructed from Equation (6)] per alignment medium need to be estimated to describe the alignment of the protein in each of the anisotropic media. Therefore, utilization of RDC data from n alignment media requires the estimation of 5n parameters. Due to the difficulty in estimating these parameters, the utility of RDC data from multiple alignment media has been limited in the past. However, recent developments [55,65] have demonstrated the possibility of accurately reconstructing 5n-3 of the needed parameters from analysis of the unassigned RDC data in the absence of structural information. The remaining three parameters, namely the α, β and γ of the molecular frame with respect to the principal alignment frame of the first order tensor [22,55,65] can be obtained via a grid search. Figure 15 illustrates the results of the 2D-RDC analysis method in estimating the relative order tensors for the unknown protein Pf2048.1. Section 4.11 provides the details of the proposed approach and how the listed order tensor in Table 12 is used throughout our analyses.

**Table 12:** Order tensors of Pf2048.1 estimated from 2D-RDC analysis using unassigned RDC data from two alignment media (Phage and PEG).

	Sxx	Sxy	Sxz	Syy	Syz	Da	R
***M1(Phage)***	2.04E−04	0.00E+00	0.00E+00	7.11E−04	0.00E+00	−10.8	0.55
***M2 (PEG)***	−9.14E−04	1.71E−05	1.61E−04	−8.55E−05	3.89E−04	−13.03	0.65

## 5. Conclusions

Computational modeling tools have made significant advances in recent years including structure determination of proteins with less than 30% sequence identity to any existing protein structure. Despite these advances, confidence in computationally modeled structures remains low especially when the ensemble of modeled structures lacks any distinct convergence in structure. As we have shown in Section 5.3, even in the presence of consistency in modeled structures, it is likely that one of the structures is a significantly better representation of the native structure than others. Interpretation of unassigned RDC data by 2D-PDPA can be very instrumental in ranking these structures and, therefore, increasing the value of computational modeling tools as another acceptable avenue of structure determination other than NMR and X-ray crystallography.

Based on the data reported by the Protein DataBank [as shown in Figure 1(b)] it can be indisputably concluded that a large portion of proteins (more than 90%) that are characterized by the general community of investigators are redundant structures. The structure determination protocols as commonly practiced do not distinguish between novel or common protein structures and therefore lead to a fixed cost of structure determination. Application of 2D-PDPA at a stage before undertaking NMR resonance assignments can identify redundant structures and give the option of halting structure determination before additional costs are incurred. It stands to reason that the cost of structure determination should be proportional to the novelty of the target protein. In Section 5.1 we have demonstrated the success of 2D-PDPA in identification of the fittest structure in a large-scale application. In addition, in Section 0 we have demonstrated success of 2D-PDPA in identification of the closest modeled structure to the native structure of Pf2048.1. Based on these results it is easy to envision a protocol where novelty of a structure is determined as the first step to structure determination before commitment of the full spectrum of data acquisition or crystallization experiments. If the unknown protein is determined to be a common protein, then computational modeling tools followed by proper ranking and validation of the modeled structures can be deployed as a viable and cost effective method of structure determination. If the unknown protein is deemed to be novel, then it can be subjected to a full experimental method of structure determination. This proposed structure determination approach could lead to a significant reduction in the average cost of structure determination while helping to identify novel structures for initiatives such as the Protein Structure Initiative or Structural Genomics Initiatives.

## References

[1] Grant A., Lee D., Orengo C. (2004). Progress towards mapping the universe of protein folds. Genome Biol..

[2] Fukunaga K. (1990). Introduction to Statistical Pattern Recognition.

[3] Greshenfeld N.A. (1998). The Nature of Mathematical Modeling.

[4] Quinn M.J., Flomenhoft M.L., Jones E.A., Flomenhoft M.L. (2003). Parallel Programming in C with MPI and OpenMP.

[5] Zhang Y. (2008). I-TASSER server for protein 3D structure prediction. BMC Bioinforma..

[6] Dunbrack R.L. (2006). Sequence comparison and protein structure prediction. Curr. Opin. Struct. Biol..

[7] Zhang Y. (2007). Template-based modeling and free modeling by I-TASSER in CASP7. Proteins.

[8] Adams M.W.W., Dailey H.A., Delucas L.J., Luo M., Prestegard J.H., Rose J.P., Wang B.C. (2003). The southeast collaboratory for structural genomics: A high-throughput gene to structure factory. Acc. Chem. Res..

[9] Brenner S.E., Levitt M. (2000). Expectations from structural genomics. Protein Sci..

[10] Service R. (2005). Structural biology - Structural genomics, round 2. Science.

[11] Berman H.M., Westbrook J., Feng Z., Gilliland G., Bhat T.N., Weissig H., Shindyalov I.N., Bourne P.E. (2000). The protein data bank. Nucleic Acids Res..

[12] Wu S., Skolnick J., Zhang Y. (2007). Ab initio modeling of small proteins by iterative TASSER simulations. BMC Biol..

[13] Kim D.E., Chivian D., Baker D. (2004). Protein structure prediction and analysis using the Robetta server. Nucleic Acids Res..

[14] Vassar R., Citron M. (2000). AÎ2-generating enzymes: Recentadvances in Î2- and Î3-secretase research. Neuron.

[15] Murzin A.G., Brenner S.E., Hubbard T., Chothia C. (1995). SCOP - a structural classification of proteins database for the investigation of sequences and structures. J. Mol. Biol..

[16] Orengo C.A., Michie A.D., Jones S., Jones D.T., Swindells M.B., Thornton J.M. (1997). CATH - a hierarchic classification of protein domain structures. Structure.

[17] Miao X., Waddell P.J., Valafar H. (2008). TALI: Local alignment of protein structures using backbone torsion angles. J. Bioinforma. Comput. Boil..

[18] Shealy P., Valafar H. (2012). Multiple structure alignment with msTALI. BMC Bioinforma..

[19] Shen Y., Vernon R., Baker D., Bax A. (2009). De novo protein structure generation from incomplete chemical shift assignments. J. Biomol. NMR.

[20] Rohl C.A., Baker D. (2002). De novo determination of protein backbone structure from residual dipolar couplings using Rosetta. J. Am. Chem. Soc..

[21] Valafar H., Prestegard J.H. (2003). Rapid classification of a protein fold family using a statistical analysis of dipolar couplings. Bioinformatics (Oxford, England).

[22] Bansal S., Miao X., Adams M.W.W., Prestegard J.H., Valafar H. (2008). Rapid classification of protein structure models using unassigned backbone RDCs and probability density profile analysis (PDPA). J. Magn. Reson..

[23] Azurmendi H.F., Martin-Pastor M., Bush C.A. (2002). Conformational studies of Lewis X and Lewis A trisaccharides using NMR residual dipolar couplings. Biopolymers.

[24] Adeyeye J., Azurmendi H.F., Stroop C.J.M., Sozhamannan S., Williams A.L., Adetumbi A.M., Johnson J.A., Bush C.A. (2003). Conformation of the hexasaccharide repeating subunit from the Vibrio cholerae O139 capsular polysaccharide. Biochemistry.

[25] Tian F., Al-Hashimi H.M., Craighead J.L., Prestegard J.H. (2001). Conformational analysis of a flexible oligosaccharide using residual dipolar couplings. J. Am. Chem. Soc..

[26] Tjandra N., Tate S., Ono A., Kainosho M., Bax A. (2000). The NMR structure of a DNA dodecamer in an aqueous dilute liquid crystalline phase. J. Am. Chem. Soc..

[27] Vermeulen A., Zhou H., Pardi A. (2000). Determining DNA global structure and DNA bending by application of NMR residual dipolar couplings. J. Am. Chem. Soc..

[28] Al-Hashimi H.M., Gorin A., Majumdar A., Gosser Y., Patel D.J. (2002). Towards structural Genomics of RNA: Rapid NMR resonance assignment and simultaneous RNA tertiary structure determination using residual dipolar couplings. J. Mol. Biol..

[29] Al-Hashimi H.M., Gosser Y., Gorin A., Hu W., Majumdar A., Patel D.J. (2002). Concerted motions in HIV-1 TAR RNA may allow access to bound state conformations: RNA dynamics from NMR residual dipolar couplings. J. Mol. Biol..

[30] Assfalg M., Bertini I., Turano P., Grant Mauk A., Winkler J.R., Gray H.B. (2003). 15N-1H Residual dipolar coupling analysis of native and alkaline-K79A Saccharomyces cerevisiae cytochrome c. Biophys. J..

[31] Andrec M., Du P., Levy R.M. (2001). Protein backbone structure determination using only residual dipolar couplings from one ordering medium. J. Biomol. NMR.

[32] Delaglio F., Kontaxis G., Bax A. (2000). Protein structure determination using molecular fragment replacement and NMR dipolar couplings. J. Am. Chem. Soc..

[33] Prestegard J.H., Mayer K.L., Valafar H., Benison G.C. (2005). Determination of protein backbone structures from residual dipolar couplings. Methods Enzymol..

[34] Valafar H., Mayer K., Bougault C., LeBlond P., Jenney F.E., Brereton P.S., Adams M., Prestegard J.H. (2005). Backbone solution structures of proteins using residual dipolar couplings: Application to a novel structural genomics target. J. Struct. Funct. Genomics.

[35] Shealy P., Liu Y., Simin M., Valafar H. (2011). Backbone resonance assignment and order tensor estimation using residual dipolar couplings. J. Biomol. NMR.

[36] Jung Y.-S.S., Sharma M., Zweckstetter M. (2004). Simultaneous assignment and structure determination of protein backbones by using NMR dipolar couplings. Angew. Chem. Int. Ed. Engl..

[37] Marassi F.M., Opella S.J. (2002). Simultaneous resonance assignment and structure determination in the solid-state NMR spectrum of a membrane protein in lipid bilayers. Biophys. J..

[38] Langmead C.J., Donald B.R. (2004). An expectation/maximization nuclear vector replacement algorithm for automated NMR resonance assignments. J. Biomol. NMR.

[39] Bernadó P., Blackledge M. (2004). Local dynamic amplitudes on the protein backbone from dipolar couplings: Toward the elucidation of slower motions in biomolecules. J. Am. Chem. Soc..

[40] Bouvignies G., Bernadó P., Meier S., Cho K., Grzesiek S., Brüschweiler R., Blackledge M. (2005). Identification of slow correlated motions in proteins using residual dipolar and hydrogen-bond scalar couplings. Proc. Natl. Acad. Sci. USA.

[41] Bryson M., Tian F., Prestegard J.H., Valafar H. (2008). REDCRAFT: A tool for simultaneous characterization of protein backbone structure and motion from RDC data. J. Magn. Reson..

[42] Andrec M., Harano Y., Jacobson M.P., Friesner R.A., Levy R.M. (2002). Complete protein structure determination using backbone residual dipolar couplings and sidechain rotamer prediction. J. Struct. Funct. Genomics.

[43] Meiler J., Peti W., Griesinger C. (2000). DipoCoup: A versatile program for 3D-structure homology comparison based on residual dipolar couplings and pseudocontact shifts. J. Biomol. NMR.

[44] Tian F., Valafar H., Prestegard J.H. (2001). A dipolar coupling based strategy for simultaneous resonance assignment and structure determination of protein backbones. J. Am. Chem. Soc..

[45] Marassi F.M., Opella S.J. (2003). Simultaneous assignment and structure determination of a membrane protein from NMR orientational restraints. Protein Sci..

[46] Prestegard J.H., Bougault C.M., Kishore A.I. (2004). Residual dipolar couplings in structure determination of biomolecules. Chem. Rev..

[47] Bertone P. (2001). SPINE: an integrated tracking database and data mining approach for identifying feasible targets in high-throughput structural proteomics. Nucleic Acids Res..

[48] Jones E.Y., Davis S.J., Williams A.F., Harlos K., Stuart D.I. (1992). Crystal structure at 2.8 A resolution of a soluble form of the cell adhesion molecule CD2. Nature.

[49] Murray A.J., Head J.G., Barker J.J., Brady R.L. (1998). Engineering an intertwined form of CD2 for stability and assembly. Nat. Struct. Biol..

[50] Valafar H., Bryson M., Miao X., Shealy P., Mukhopadhyay R., Yandle R., Simin M., Fahim A., Irausquin S.J. ValafarLab web page. http://ifestos.cse.sc.edu.

[51] Ulrich E.L., Akutsu H., Doreleijers J.F., Harano Y., Ioannidis Y.E., Lin J., Livny M., Mading S., Maziuk D., Miller Z. (2008). BioMagResBank. Nucleic Acids Res..

[52] Doreleijers J.F., Mading S., Maziuk D., Sojourner K., Yin L., Zhu J., Markley J.L., Ulrich E.L. (2003). BioMagResBank database with sets of experimental NMR constraints corresponding to the structures of over 1400 biomolecules deposited in the Protein Data Bank. J. Biomol. NMR.

[53] Ulmer T.S., Ramirez B.E., Delaglio F., Bax A. (2003). Evaluation of backbone proton positions and dynamics in a small protein by liquid crystal NMR spectroscopy. J. Am. Chem. Soc..

[54] Cornilescu G., Marquardt J.L., Ottiger M., Bax A. (1998). Validation of protein structure from anisotropic carbonyl chemical shifts in a dilute liquid crystalline phase. J. Am. Chem. Soc..

[55] Mukhopadhyay R., Miao X., Shealy P., Valafar H. (2009). Efficient and accurate estimation of relative order tensors from lambda-maps. J. Magn. Reson..

[56] Doreleijers J.F., Raves M.L., Rullmann T., Kaptein R. (1999). Completeness of NOEs in protein structure: A statistical analysis of NMR data. J. Biomol. NMR.

[57] Bau R., Rees D.C., Kurtz D.M., Scott R.A., Huang H., Adams M.W.W., Eidsness M.K. (1998). Crystal structure of rubredoxin from Pyrococcus furiosus at 0.95 Å resolution, and the structures of N-terminal methionine and formylmethionine variants of Pf Rd. Contributions of N-terminal interactions to thermostability. J. Biol. Inorg. Chem..

[58] Valafar H., Prestegard J.H. (2004). REDCAT: A residual dipolar coupling analysis tool. J. Magn. Reson..

[59] Word J.M., Lovell S.C., Richardson J.S., Richardson D.C. (1999). Asparagine and glutamine: Using hydrogen atom contacts in the choice of side-chain amide orientation. J. Mol. Biol..

[60] Holm L., Sander C. (1994). The FSSP database of structurally aligned protein fold families. Nucleic Acids Res..

[61] Prestegard J.H., Kishore A.I. (2001). Partial alignment of biomolecules: An aid to NMR characterization. Curr. Opin. Chem. Biol..

[62] Otting G., Rückert M., Levitt M. H., Moshref A. (2000). NMR experiments for the sign determination of homonuclear scalar and residual dipolar couplings. J. Biomol. NMR.

[63] Ottiger M., Bax A. (1998). Determination of relative N−H N, N−C‘, C α −C‘, and C α −H α effective bond lengths in a protein by NMR in a dilute liquid crystalline phase. J. Am. Chem. Soc..

[64] Delaglio F., Grzesiek S., Vuister G.W., Zhu G., Pfeifer J., Bax A. (1995). NMRPipe: A multi- dimensional spectral processing system based on UNIX pipes. J. Biomol. NMR.

[65] Miao X., Mukhopadhyay R., Valafar H., MR&VH M.X. (2008). Estimation of relative order tensors, and reconstruction of vectors in space using unassigned RDC data and its application. J. Magn. Reson..

[66] Saupe A., Englert G. (1963). High-resolution nuclear magnetic resonance spectra of orientated molecules. Phys. Rev. Lett..

[67] Blackledge M. (2005). Recent progress in the study of biomolecular structure and dynamics in solution from residual dipolar couplings. Prog. Nuclear Magn. Reson. Spectrosc..

[68] Baran M.C., Huang Y.J., Moseley H.N.B., Montelione G.T. (2004). Automated analysis of protein NMR assignments and structures. Chem. Rev..

[69] Mayer K.L., Qu Y., Bansal S., LeBlond P.D., Jenney F.E., Brereton P.S., Adams M.W.W., Xu Y., Prestegard J.H. (2006). Structure determination of a new protein from backbone-centered NMR data and NMR-assisted structure prediction. Proteins Struct. Funct. Bioinf..

[70] Wang X., Bansal S., Jiang M., Prestegard J.H. (2008). RDC-assisted modeling of symmetric protein homo-oligomers. Protein Sci..

[71] Serfiotis-Mitsa D., Herbert A.P., Roberts G.A., Soares D.C., White J.H., Blakely G.W., Uhrín D., Dryden D.T.F. (2010). The structure of the KlcA and ArdB proteins reveals a novel fold and antirestriction activity against Type I DNA restriction systems *in vivo* but not *in vitro*. Nucleic Acids Res..

[72] Wang J., Walsh J.D., Kuszewski J., Wang Y.-X. (2007). Periodicity, planarity, and pixel (3P): A program using the intrinsic residual dipolar coupling periodicity-to-peptide plane correlation and phi/psi angles to derive protein backbone structures. J. Magn. Reson..

[73] Stelzer A.C., Frank A.T., Bailor M.H., Andricioaei I., Al-Hashimi H.M. (2009). Constructing atomic-resolution RNA structural ensembles using MD and motionally decoupled NMR RDCs. Methods.

[74] Croy J.E., Wuttke D.S. (2009). Insights into the dynamics of specific telomeric single-stranded DNA recognition by Pot1pN. J. Mol. Biol..

[75] Latham M.P., Hanson P., Brown D.J., Pardi A. (2008). Comparison of alignment tensors generated for native tRNA(Val) using magnetic fields and liquid crystalline media. J. Biomol. NMR.

[76] Bailor M.H., Musselman C., Hansen A.L., Gulati K., Patel D.J., Al-Hashimi H.M. (2007). Characterizing the relative orientation and dynamics of RNA A-form helices using NMR residual dipolar couplings. Nat. Protoc..

[77] Mackeen M.M., Almond A., Deschamps M., Cumpstey I., Fairbanks A.J., Tsang C., Rudd P.M., Butters T.D., Dwek R.A., Wormald M.R. (2009). The conformational properties of the Glc3Man unit suggest conformational biasing within the chaperone-assisted glycoprotein folding pathway. J. Mol. Biol..

[78] Zhuang T., Lee H.-S., Imperiali B., Prestegard J.H. (2008). Structure determination of a Galectin-3-carbohydrate complex using paramagnetism-based NMR constraints. Protein Sci..

[79] Zhuang T., Leffler H., Prestegard J.H. (2006). Enhancement of bound-state residual dipolar couplings: Conformational analysis of lactose bound to Galectin-3. Protein Sci..

[80] Prestegard J.H., Yi X., Vliegenthar J.F.G., Woods R.J. (2006). NMR Spectroscopy and Computer Modeling of Carbohydrates.

[81] Teriete P., Franzin C.M., Choi J., Marassi F.M. (2007). Structure of the Na,K-ATPase regulatory protein FXYD1 in micelles. Biochemistry.

[82] Gong X.-M., Franzin C., Thai K., Yu J., Marassi F.M. (2007). Nuclear magnetic resonance structural studies of membrane proteins in micelles and bilayers. Methods Mol. Biol..

[83] Valentine K.G., Pometun M.S., Kielec J.M., Baigelman R.E., Staub J.K., Owens K.L., Wand A.J. (2006). Magnetic susceptibility-induced alignment of proteins in reverse micelles. J. Am. Chem. Soc..

[84] Franzin C.M., Yu J., Thai K., Choi J., Marassi F.M. (2005). Correlation of gene and protein structures in the FXYD family proteins. J. Mol. Boil..

[85] Bhattacharya A., Kurochkin A.V., Yip G.N.B., Zhang Y., Bertelsen E.B., Zuiderweg E.R.P. (2009). Allostery in Hsp70 chaperones is transduced by subdomain rotations. J. Mol. Biol..

[86] Seidel R.D., Zhuang T., Prestegard J.H. (2007). Bound-state residual dipolar couplings for rapidly exchanging ligands of His-tagged proteins. J. Am. Chem. Soc..

[87] Prestegard J.H., Al-Hashimi H.M., Tolman J.R. (2000). NMR structures of biomolecules using field oriented media and residual dipolar couplings. Q. Rev. Biophys..

